# U-Infuse: Democratization of Customizable Deep Learning for Object Detection

**DOI:** 10.3390/s21082611

**Published:** 2021-04-08

**Authors:** Andrew Shepley, Greg Falzon, Christopher Lawson, Paul Meek, Paul Kwan

**Affiliations:** 1 School of Science and Technology, University of New England, Armidale, NSW 2350, Australia; greg.falzon@flinders.edu.au (G.F.); christopher.lawson@uon.edu.au (C.L.); 2 College of Science and Engineering, Flinders University, Adelaide, SA 5001, Australia; 3 Vertebrate Pest Research Unit, NSW Department of Primary Industries, P.O. Box 530, Coffs Harbour, NSW 2450, Australia; paul.meek@dpi.nsw.gov.au; 4 School of Environmental and Rural Science, University of New England, Armidale, NSW 2350, Australia; 5 School of IT and Engineering, Melbourne Institute of Technology, Melbourne, VIC 3000, Australia; pkwan@mit.edu.au

**Keywords:** animal identification, artificial intelligence, camera-trap images, camera trapping, deep convolutional neural networks, deep learning, environmental software, wildlife ecology, wildlife monitoring, ecological object detection

## Abstract

Image data is one of the primary sources of ecological data used in biodiversity conservation and management worldwide. However, classifying and interpreting large numbers of images is time and resource expensive, particularly in the context of camera trapping. Deep learning models have been used to achieve this task but are often not suited to specific applications due to their inability to generalise to new environments and inconsistent performance. Models need to be developed for specific species cohorts and environments, but the technical skills required to achieve this are a key barrier to the accessibility of this technology to ecologists. Thus, there is a strong need to democratize access to deep learning technologies by providing an easy-to-use software application allowing non-technical users to train custom object detectors. U-Infuse addresses this issue by providing ecologists with the ability to train customised models using publicly available images and/or their own images without specific technical expertise. Auto-annotation and annotation editing functionalities minimize the constraints of manually annotating and pre-processing large numbers of images. U-Infuse is a free and open-source software solution that supports both multiclass and single class training and object detection, allowing ecologists to access deep learning technologies usually only available to computer scientists, on their own device, customised for their application, without sharing intellectual property or sensitive data. It provides ecological practitioners with the ability to (i) easily achieve object detection within a user-friendly GUI, generating a species distribution report, and other useful statistics, (ii) custom train deep learning models using publicly available and custom training data, (iii) achieve supervised auto-annotation of images for further training, with the benefit of editing annotations to ensure quality datasets. Broad adoption of U-Infuse by ecological practitioners will improve ecological image analysis and processing by allowing significantly more image data to be processed with minimal expenditure of time and resources, particularly for camera trap images. Ease of training and use of transfer learning means domain-specific models can be trained rapidly, and frequently updated without the need for computer science expertise, or data sharing, protecting intellectual property and privacy.

## 1. Introduction

The use of camera trap image analysis in biodiversity management is one of the primary means by which ecological practitioners monitor wildlife [1,2,3,4,5], obtain species distribution [6], perform population estimates [7,8,9,10] and observe animal behavioural patterns [11]. However, this results in millions of images being captured, which must be processed. This is a time and resource expensive task, often manually undertaken by ecologists, which has given rise to a strong need for automation [12]. This has triggered significant interest in deep learning-based image processing solutions [13,14,15,16,17,18,19,20].

### Related Works

Wildlife Insights [21] provides an online cloud-based service allowing practitioners to upload camera trap images to a Google Cloud-based platform which filters empty images and performs classification on 614 species. Similarly, Microsoft AI for Earth Camera Trap API [22] uses a cloud-based system to perform object detection on large quantities of camera trap images using MegaDetector. It can be used in conjunction with TimeLapse2 [23] and Camelot [24], however inference is only available for separation of empty/non-empty images, and detection of limited classes. Other cloud-based inference systems include Project Zamba [25], which is a Python toolkit specific to African species. Although these services facilitate the task of camera trap image processing, users do not have the option to train models on their own images, nor can they use the services on their own device, without sharing their image data. Furthermore, models are often not sufficiently location invariant to be used with high confidence, limiting their widespread usage [26].

Alternatives to cloud-based services include ClassifyMe [20], which upon registration provides offline, on-device access to more than five YOLOv2 [27] models trained on publicly available camera trap datasets. However, these models are highly optimised for specific environments, meaning they do not generalise well to unseen environments, and users are unable to train their own models. Another alternative is Machine Learning for Wildlife Image Classification (MLWIC) [19], which allows the development of custom models using the R Programming Language [28]. However, this requires technical knowledge and a large investment of time and resources into model development. Similarly, Camera-Trap-Classifier [17], which is an experimental camera trap object detector, requires knowledge of Unix, limiting its adoption by non-technical ecological practitioners.

One significant issue faced by all object detection solutions is the lack of location invariance of deep learning models, and their inability to generalise to unseen environments [18,26,29,30]. Due to the inherent difficulty represented by high occlusion, illumination, high object density, camouflage, movement and poor data quality usually featured in camera trap images, the development of high precision camera trap object detectors capable of generalisation to any environment is a challenge that is yet to be achieved [14]. This means they lack sufficient accuracy to be deployed in domains not included in the training data [18,26]. Transfer learning has been used to improve ability to generalise [17] however optimal performance can only be attained if the ecological practitioner has access to a model trained on their own data. Furthermore, development and deployment of such models requires specialised computer science skills [20].

These needs are addressed by U-Infuse, which is a novel software application that provides a means by which ecological practitioners can easily train their own deep learning object detectors. Significant investment of time in the technical programming and specialised artificial intelligence domain knowledge are not required to develop custom models. U-Infuse is designed to enable ecologists to train models according to the requirements of any given project. They may wish to train location invariant object detection models on publicly available data and camera trap images according to the ‘infusion’ methodology proposed by [31], or highly domain-specific, location variant training of models optimised for specific locations. This flexibility in the way U-Infuse can be used allows practitioners to process image data in-house, at their own pace according to their needs, removing the need to allocate significant time and resources to upload, save and share data with service providers, which is a drawback of existing solutions.

## 2. The U-Infuse Application

U-Infuse is a free, open-source software application supported by Windows 10, and Linux operating systems, with source code provided to extend it to other operating systems. The U-Infuse app is developed in the Python 3 programming language with bindings to core Python-based machine learning frameworks and image processing facilities along with a Qt5 Graphical User Interface (GUI). It has been verified on Windows 10 Home and Professional, Ubuntu 18.04–20.04, and CentOS. It may be used on other systems within a virtual machine. Copying, distribution and modification of U-Infuse source code is encouraged. Accordingly, U-Infuse is distributed under the terms of a GNU General Public License (https://www.gnu.org/licenses/gpl-3.0.en.html, accessed on 1 April 2021).

For best user experience, practitioners are encouraged to use the downloadable executable file for installation, on a Windows 10 system. The simple-to-use GUI allows users to auto-annotate training images, custom train their own object detectors using RetinaNet [32], and perform inference using pretrained or custom models on custom datasets. GUI Performance has been verified for large datasets, containing approximately 10,000 s of images. U-Infuse is available online at GitHub (https://github.com/u-infuse/u-infuse, accessed on 1 April 2021). All U-Infuse functionalities are also provided via Python scripts and Jupyter Notebooks, which contain the U-Infuse pipeline that can be used as is, incorporated into, or adapted to any project on any platform. Whilst the GUI is appropriate for workstations processing thousands of images, the Jupyter Notebooks allow the U-Infuse functionalities to be extended to datasets of any size, on high performance computing systems. All upgrades, demonstrations and tutorials are available on the corresponding GitHub Wiki.

Installation of U-Infuse is straightforward and is achieved either from source code or via a downloadable binary executable file complete with an installation wizard (Windows only). Software dependencies include RetinaNet [32], TensorFlow [33], OpenCV and Python 3. The installation script automatically incorporates these dependencies within the installation. Model training via the Graphical Processor Unit is facilitated for CUDA supported hardware and requires cuDNN and the CUDA development toolkit (which must be installed by the user).

### Functionalities

**Figure 1:**
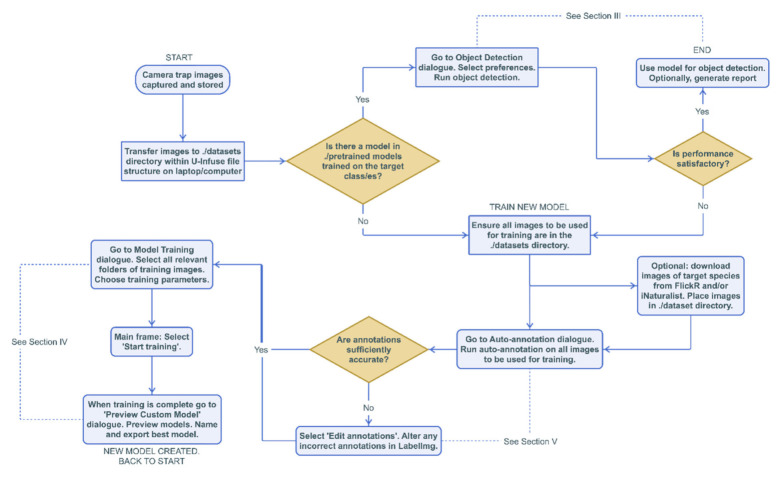
Sample workflow diagram demonstrating pipeline useability. Please refer to [30] for best practices when using FlickR and iNaturalist (FiN) images.

U-Infuse provides users with a complete object detection pipeline, supporting image annotation, object detector training and inference capabilities. It uses as input image datasets supporting all of the most commonly used image formats including PNG, JPEG and TIFF, and optional corresponding annotation files (.xml format). All training scripts and files are contained within the U-Infuse installation, or generated by user-initiated training, inference or annotation processes. See Figure 1 for the workflow diagram.

## 3. Animal Detection and Classification Using Default Models

The U-Infuse installation comes with six pretrained RetinaNet object detector models. Detailed information about these models is provided in Table 1. These models are provided to be used as pretrained models for user-controlled transfer learning, for inference and demonstration purposes.

**Figure 2:**
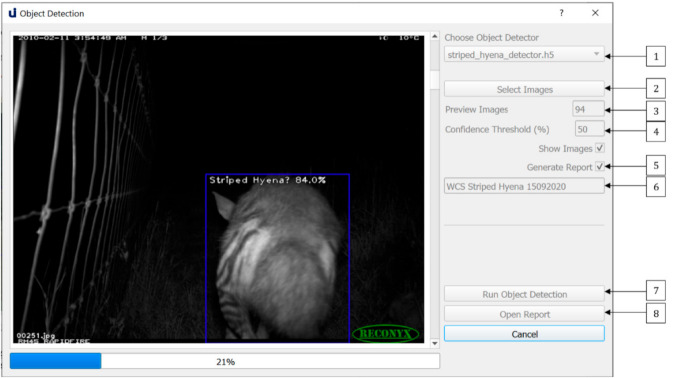
The Object Detection dialogue allows users to perform object detection on a dataset of images, optionally generating an object detection report.

These models can be used for camera trap image classification via the *Object Detection* dialogue, shown in Figure 2. Users can select the model of their choice via the dropdown list (1). The chosen model will perform object detection on all the images contained in the chosen image folder (2). Users may optionally limit the number of images on which inference is conducted via the option at (3), with the default being the number of images in the chosen folder. After choosing the level of desired accuracy via the confidence threshold (4), they may elect to show images while inference is running and generate an object detection report (5).

**Figure 3:**
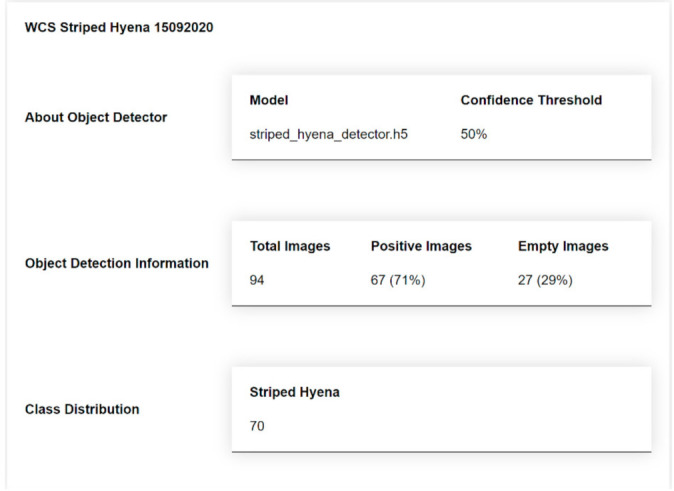
A sample summary report generated by running inference on a dataset of images. The report provides a summary of object detection information, including class distribution, number and percentage of positive images compared to empty images.

**Figure 4:**
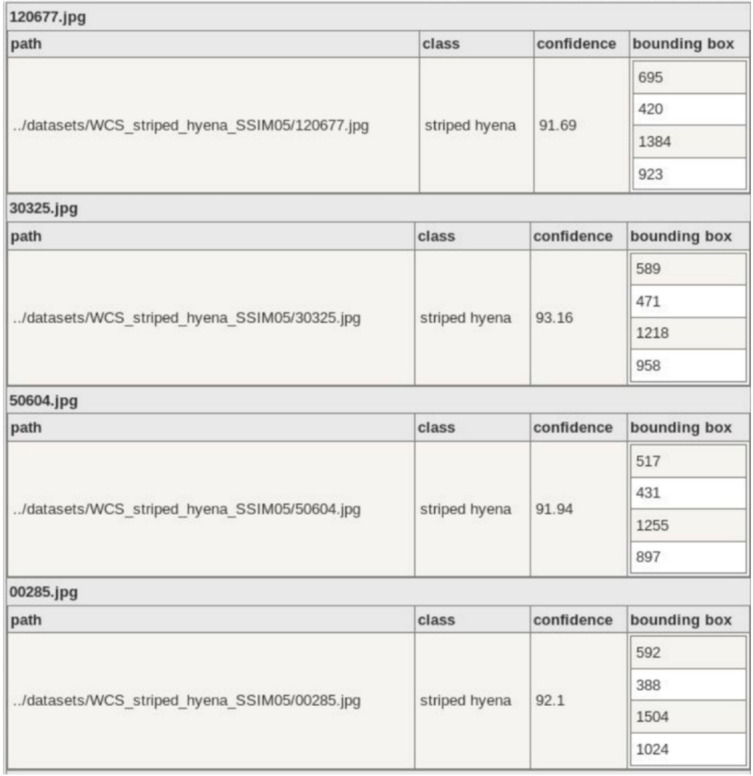
A sample of the detailed report, which shows per image data, including class label, classification confidence and bounding box data for each object. This preview is generated using json2table.com.

If they choose to generate an inference report, they must provide a name for their report (6). A summary and detailed report will be generated in JSON format and saved in the *./reports* directory. The summary report contains information about the model used, the dataset, and the object detection output, such as species distribution and number of empty images. The detailed report contains object detection data for each image, for example, number of objects, class, confidence of detections and bounding box coordinates. Users can optionally generate a JSON file containing references to each empty image. A sample of the Summary Report is shown in Figure 3, and a sample of the Detailed Report is shown in Figure 4. The Summary Report can be opened via the open at (8).

## 4. Custom Model Training Using FlickR and Camera Trap Image Infusion

If the results attained by the default models are not sufficiently accurate for the user’s purposes, or the models are not sufficiently specific to the user’s domain, they may elect to custom train their own model or models. They may choose to use publicly available images such as FlickR and iNaturalist (FiN) images, infused with camera trap images as proposed by [31] or they may alternatively train using images from any source, including publicly available images, and/or their own trap images. It is strongly recommended that users use negative sampling when training custom models because negative sampling has been shown to significantly improve the ability of a model to discriminate between positive and negative objects [34]. Negative sampling refers to the inclusion of unannotated non-target objects, which share similarities with the target class, in the training set. For example, if an object detector Is being trained to detect feral dogs, negative samples of similar animals such as foxes and feral cats should be included in training.

### 4.1. Dataset and Classes

All datasets to be used for training should be placed in the ‘datasets’ directory within the U-Infuse base directory. Users may add datasets of their choice to this directory or download U-Infuse FlickR annotated datasets from the U-Infuse GitHub page. The U-Infuse dataset repository contains 35 freely available single class datasets. Users may choose to use these datasets, or other publicly available datasets, or private datasets such as project specific camera trap images. User images are not shared, or accessible outside the user’s network or device, ensuring protection of intellectual property and privacy. All bounding box annotations must be placed in the ‘annotations’ directory, also within the U-Infuse base directory. Users must ensure that any custom datasets are accompanied by corresponding annotation files, which must be placed in the ‘annotations’ directory in a folder with the same name as the corresponding image dataset. For example, if the user adds the image dataset ‘New England Jan’ to ‘datasets’ they must provide annotations in a folder named ‘New England Jan’ in the ‘annotations’ directory. These annotations must be in PASCAL VOC format. Alternative annotation formats including YOLO may be supported in future releases. If a user does not have annotations, they may auto-annotate their images within U-Infuse, as discussed in Section 6.

**Figure 5:**
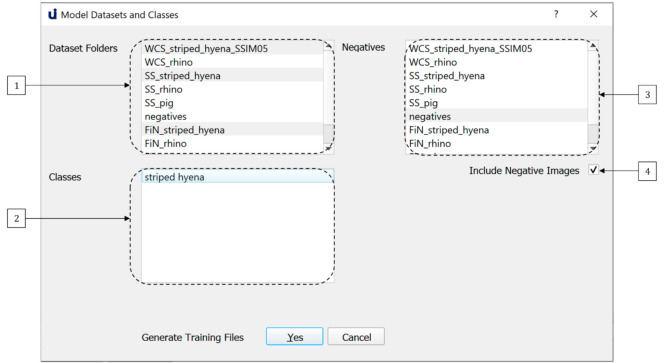
The Model Training dialogue allows users to custom train models on datasets and classes of their choice.

Once the user has ensured that their image and annotation folders are located correctly in datasets and annotations, they may proceed to the training process, which can be initiated via the Training Datasets and Classes dialogue shown in Figure 5. Users must select one or more datasets for inclusion in training from the list of available datasets shown in the combo-box denoted by (1).

Once the user has selects one or more datasets, the list of classes available for training within those datasets is shown in the combo-box denoted by (2). If a dataset is deselected, classes specific to that dataset can no longer be chosen. U-Infuse supports both single class and multi-class training, meaning the user can select one or more classes for inclusion in training from (2). For example, the user may select five datasets, containing a total of twelve classes, but they may choose to only train on three of the available classes. They can then select one or more datasets from the combobox denoted by (3) for negative sampling. Alternatively, they can select the option to use all other classes for negative sampling. They may elect not to use negative sampling (4) however this is not recommended.

### 4.2. Training

After dataset and class selection, the user may proceed to the training phase. Training requires the use of a Graphical Processing Unit (GPU) supporting CUDA [35] due to the computationally expensive nature of training deep neural networks. Deep learning is a resource-intensive process that cannot be effectively achieved using a standard CPU. GPUs are specialised hardware used to process images, as they can handle large amounts of data, and support parallel processing. It is worth noting that U-Infuse does not currently support training without pre-initialised network weights, which requires thousands to millions of images to achieve acceptable results, due to random initialisation of weights. Instead, U-Infuse provides capability for modifying pre-trained network weights using transfer learning, which requires comparatively minimal data and computational cost to develop effective deep learning models [16,17].

**Figure 6:**
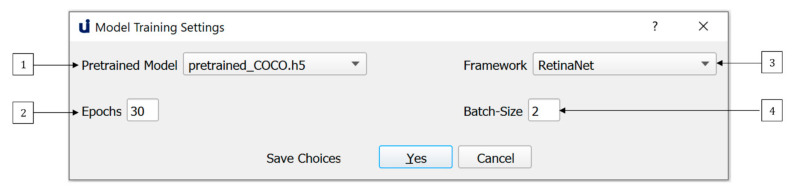
Default training parameters are provided however users may elect to modify these depending on their GPU capabilities and dataset size.

U-Infuse features default training parameters established within our research program. They can be modified via the Model Training Settings Dialogue shown in Figure 6. Firstly, the user must select a pretrained model (1). U-Infuse provides 6 pretrained models, one of which can be selected as the backbone for further model training. User provided or trained models can also be used as the basis for further model training, if placed in the *./pretrained_models* directory. Training time will not be significantly affected by the user’s selection of pretrained models. Pretrained models should be chosen based on the user’s target species. For example, if they wish to create a vehicle detector, they should choose the pretrained_COCO model as their backbone, as it has been trained on cars and trucks already, so it will have learned some of the relevant features. Similarly, if the user wishes to create a macropod detector, they should choose the Australian_Multi-class pretrained model as their backbone, as it was trained on 30 Australian species, including wallabies and kangaroos.

Users may elect to modify the ‘Epochs’ (2) option. By default, training will proceed for 30 epochs, however users can increase or decrease the number of epochs, depending on the accuracy they seek. Reducing the number of epochs will reduce training time, however it may also reduce accuracy. Increasing the number of epochs means that the model is trained for longer, resulting in higher model training accuracy, but can lead to overfitting. Overfitting occurs when the network memorises the features of the training data, limiting its ability to generalise to other datasets. To avoid overfitting and allow monitoring of training, U-Infuse outputs data such as the training and validation loss, as well as Mean Average Precision (mAP) results calculated on a validation dataset after each training epoch. Note, mAP refers to the mean of the average precision (AP) scores calculated for each class. For an explanation of AP, see [36].

To use U-Infuse for training, users must have access to a GPU. As GPU capability may vary between users, the batch size used for training can be varied (4). A default batch size of 2 is provided, however users with high capability GPUs may elect to increase this value, while those with limited GPU resources may reduce the batch size to 1. The greater the batch size the more GPU memory required for training. Once the datasets, classes and training parameters have been selected, the user can proceed to generate the training scripts. Alternatively, the dialogue may be closed, with no changes saved by selecting the Cancel option. Any error or success messages are shown in the main frame output window.

**Figure 7:**
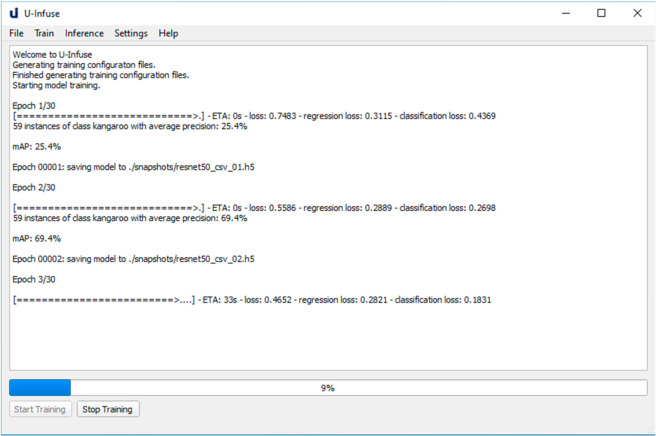
The main frame which allows users to start, stop and monitor training of custom models. Training progress can be monitored via the overall training loss, regression loss and classification loss. Generally, the lower the loss, the better the training. Model performance can also be monitored via the validation mAP, which is calculated on a subsample of the training data.

If the training configuration process is successfully completed, the Start Training option on the main frame may be selected to train a new RetinaNet model. Training will usually take several hours, depending on the dataset size, number of epochs and batch size used. During training the user will be updated by messages in the main frame, as shown in Figure 7. After each epoch, a snapshot of the model is saved in the *./snapshots* directory. A snapshot is a file containing the weights of the model after a given epoch and should not be confused with terms such as Snapshot Serengeti project.

**Figure 8:**
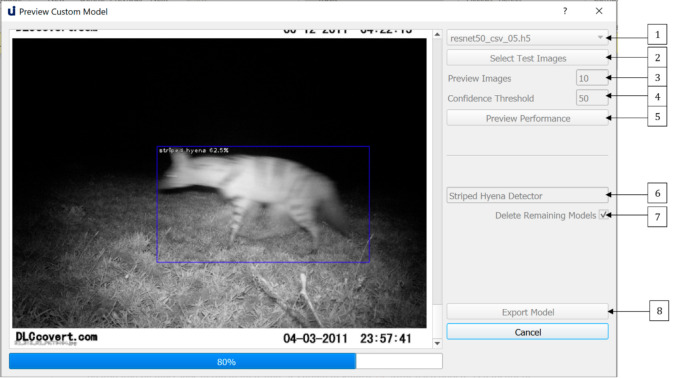
The Preview Custom Model dialogue allows the user to preview the performance of custom trained models on a small subset of randomly sampled test images. It also allows users to export chosen models for future use.

Once training is complete, the user can choose which snapshot they wish to retain as their final custom model. They can preview the performance of snapshots via the *Preview Custom Model* dialogue shown in Figure 8. They can choose snapshots via the dropdown list at (1), select their test images (2) and the number of images they wish to preview (3) as well as a level of accuracy (4). Once they run the model (5), they may name their model (6) and it (8). It is recommended that users delete all other models (7) as snapshots are large files. The exported model will be saved, to be reused via the *Object Detection* dialogue for object detection on any dataset, as described in Section 4.

## 5. Auto-Annotation and Manual Annotation Editing

**Figure 9:**
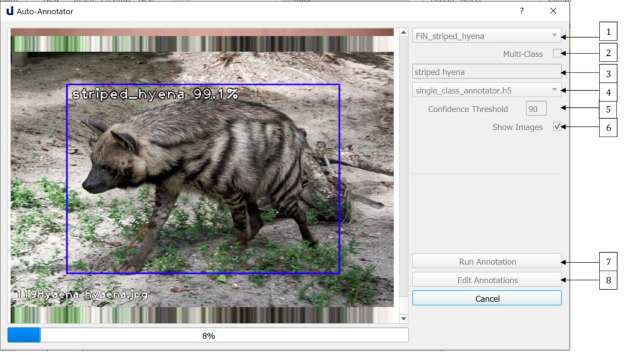
The Auto-Annotation dialogue allows users to achieve both single class and multi-class auto-annotation of images. Annotations can be edited via labelImg.

One of the most time-consuming aspects of developing deep learning-based object detectors is the annotation of training images. U-Infuse automates this process, by allowing users to employ pretrained models (object detectors), or a provided Single Class Annotator to annotate any number of images. This can be achieved via the *Auto-Annotation* dialogue shown in Figure 9. Users must add the folder/s containing the images they wish to annotate to the ‘datasets’ directory. They can then access their chosen dataset via the dropdown list (1). If the user chooses to conduct multi-class annotation (2), they must select a pretrained model from the list of available options (4). The labels used to annotate objects will be chosen by the pretrained model. Alternatively, the user can provide a single class label (3) which will be used to annotate all bounding boxes generated by the provided auto-annotator. This is very useful in cases where the user wants to annotate a dataset containing objects for which they do not have an object detector. Users can also vary the confidence threshold (5). A higher confidence threshold means less bounding boxes (potential objects) are retained, while a lower confidence threshold allows more bounding boxes to be shown, hence a greater number of potential detections are retained. Users may elect to show images (6) or not. Note, electing to show images is more computationally expensive, meaning the annotation process will usually take longer.

**Figure 10:**
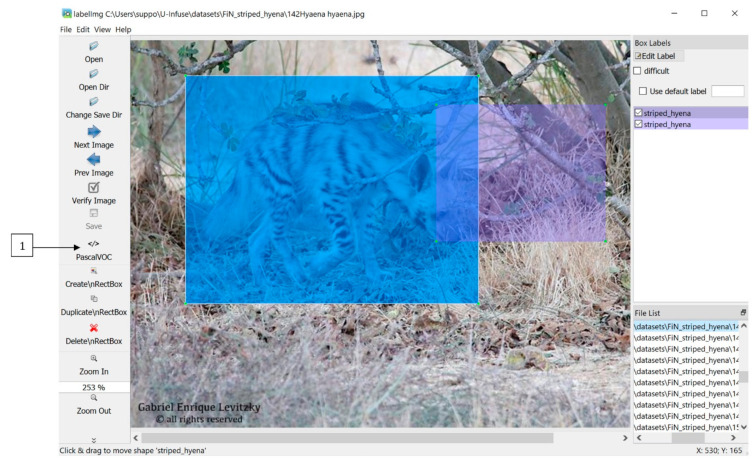
Annotations can be edited in labelImg. Bounding boxes can be added, removed or altered. Labels can also be altered.

Once the user has run the annotation model (7), they may edit these annotations (8) via labelImg [37]. LabelImg is a separate, open source and freely available app which is opened ‘Edit Annotations’ is clicked. It can be used to remove unwanted boxes, edit the position or size of boxes or add boxes around objects that were missed. Sometimes, the auto-annotator will miss objects, add extra boxes, or the boxes may not be optimal, for example, the trap image shown in Figure 10 contains an extra box which should be removed.

Bounding boxes can be added, removed or resized, and labels can be changed within the labelImg GUI as shown in Figure 10 Users must ensure they save any altered annotations in Pascal VOC format by selecting the option denoted by (1) in Figure 10. Once this process is completed, the images and corresponding annotation files can be used to train models, as described in Section 5.

## 6. Case Study: Monitoring and Managing Feral Cats

In this section, we present a brief real-world case study exemplifying the usage of U-Infuse to process camera trap images of feral cats collected as part of an extensive feral cat management program in the New England Gorges region in Northern NSW, Australia. We evaluate U-Infuse in the task of training a feral cat detector, as well as the application of its object detection functionalities for image data analysis.

### 6.1. Background

Democratizing access to deep learning model development and usage is of primary importance in the management of ecological resources, including the monitoring and management of invasive species [38]. Invasive species such as feral cats have a significant detrimental impact on natural ecosystems and native species in Australia. Every year, feral cats are thought to kill over 1 billion animals across Australia [39]. They are also responsible for transmitting diseases such as *Toxoplasma gondii*, which can cause illness and death in both native fauna and livestock [40].

Despite aspirations to control feral cat populations throughout Australia, there are currently no cost-effective methods available to managers to effectively mitigate impacts. As such, numerous Australian academic institutions, conservation groups and government agencies have prioritised the development of deep learning models capable of confidently detecting and localising feral cats in camera trap imagery. This approach provides a foundation for automated or more efficient image processing, enabling effective monitoring of feral cat populations, and under-pinning the basis of automated management tools. This technology also presents a range of tools that can be extended beyond feral cats, to a broad range of invasive species, as well as native species in Australia and internationally.

### 6.2. FiN-Infusion Training with U-Infuse

We applied U-Infuse to develop and use a feral cat detector for camera trap image processing. U-Infuse was downloaded from GitHub and installed on a CentOS Linux system with NVIDIA GV100GL [Tesla V100 PCIe 32 GB] Graphical Processing Unit (GPU). The feral cat detector was developed using the Model Training functionality within U-Infuse, as well as Auto-Annotation and model exporting features.

We used the FiN-infusion training methodology proposed by [31] to train a feral cat detector. The training and test sets are described in Table 2. Our training dataset comprised of a total of 5043 images, including 1216 positive samples (images containing cats) and 3827 negative samples (blank images, or images of other animals). We performed 22% out of sample camera trap infusion. Infusion refers to training a model on a small subset of camera trap images as well as a larger set of FiN images to improve robustness of our model to the particularities of camera trap images. Out of sample means that the infusion training images are from different sites to the test images. We chose to include a large proportion of negative samples, with focus on visually similar animals such as foxes and dogs, as well as animals commonly found around our trap sites. Negative sampling significantly reduces the number of false positives, improving the reliability and accuracy of the model.

Positive samples were auto-annotated using the U-Infuse single class auto-annotator. Approximately 15% (238 images) of annotations required correction for reasons including false negatives, or bounding boxes that were either too large or small. Annotating 1216 images of a low-density species such as feral cat usually takes a single human annotator up to 3 h. Auto-annotation using U-Infuse took only 3 min and 15 s. Correcting poorly annotated images took approximately 15 min. This represents an approx. 90% decrease in time expenditure on the annotation task. It is noteworthy that the datasets used for training were pre-sorted (as containing a feral cat, a non-target class, or no object). There was no need to annotate negative images (blanks or non-target classes) because U-Infuse automatically validates all negative samples selected prior to generating training files.

The testing datasets comprised of positive and negative images. The NE_Gorge datasets are comprised of images from the New England Gorges in Northern NSW. One dataset contains images captured in daylight, whilst the other contains infrared images captured at night. The second testing data source was the Wellington (cat) dataset available on the LILA repository [41]. It contains images of feral cats collected using 187 camera traps in various locations in Wellington, New Zealand. We tested our model on two completely different projects to ensure robustness to variations in camera trap configuration and environmental features such as vegetation and landforms.

We evaluated our model on a per image and per capture event basis. Per image evaluation is when the model correctly classifies an image as containing a cat, or not. Per capture event evaluation is when the model correctly classifies a group of images captured in a sequence as containing at least one instance of a cat. A capture event happens when a camera captures a sequence of images in a burst based on predefined user settings, when the camera motion detector is triggered. The NE_Gorge data contains capture events made up of 1 to 10 images, while the Wellington data contains 3 images per capture event.

Due to having access to a capable GPU, we modified the training settings to batch size of 8 and trained for a total of 30 epochs. The loss converged on 0.63 with a validation mAP of 93%. Total training time was 6 h and 38 min. Notably, the time taken to perform tasks outlined in this method is representative only of this application, and times taken for annotation and training will vary considerably, depending on factors such as dataset size, image quality and GPU capabilities.

### 6.3. Per Image and Per Capture Event Performance

Once training terminated, we used the Preview Custom Model dialogue to preview the performance of some of our models (one model is saved per epoch). We chose to retain the final epoch snapshot. We named this model ‘felis_catus_detector’ and exported it. This model was now in our *./pretrained* models directory for use in camera trap object detection. We then evaluated the performance of our model on the test sets described in Table 2. Results are presented in Table 3.

### 6.4. Discussion

Real world camera trap image quality is often very poor, or characterized by specific features not present in other camera trap datasets, or publicly available images. To achieve highly accurate object detection, it is therefore often necessary to train on images from the study location. This is facilitated by the use of the FiN-infusion method proposed by [30], which involves training on a combination of highly variable publicly available images, and a small subset of in-sample camera trap images collected as part of the field study to boost confidence in difficult images. U-Infuse allows ecologists to use the FiN-infusion training themselves, to train their own high accuracy object detections models without having to resort to up-skilling in computer science. It therefore represents a significant contribution in helping to overcome the problem of poor performance of generic models on difficult real-world camera trap images.

The U-Infuse Object Detection dialogue can be used to perform object detection on any number of images, with the option to generate a summary report and a detailed report (per image data). The summary report is useful in reporting the number of blank images (images not containing any animals of interest, or animals able to be detected by the model), as well as the number of images containing a cat. If U-Infuse is used for multi-class detection, the summary report would show the class distribution (calculated on a per image basis), which is a list of the different animal types detected by the model in the set of images, as well as how many instances of each animal.

We used the detailed report (per image data) to group images into capture events and report performance on a per capture event basis. To achieve this, we were required to write an algorithm to group images based on image name, which is highly dependent on the data collection method. One possible improvement to U-Infuse would be to allow for per capture event performance reporting without requiring users to post-process the detailed report. This would improve usability of the software.

A very useful feature of U-Infuse is the ease at which the object detection model can be used in conjunction with other image processing software. Thus, it may be used for one or more purposes, e.g., auto-annotation of images (no GPU required), model training (GPU required), and/or object detection (no GPU required). This flexibility means it can easily be incorporated in existing image processing pipelines, offering the benefits of automation of tasks that usually require significant human time expenditure.

## 7. Future Work and Conclusions

U-Infuse is a free and open source application which implements the location invariance methodology proposed by [31]. It democratises deep learning and AI technologies by making deep learning more accessible for ecological practitioners. Furthermore, it can be used for location invariant object detection in fields outside of ecology. Its open source nature means members of the community can contribute to its development, incorporating it and using it to complement existing software and deploying models developed to cloud-based platforms. Ecologists are encouraged to contribute their annotated camera trap images to the U-Infuse repository to contribute to the development of more powerful, location invariant object detectors.

A major constraint on the widespread deployment of AI in ecology and automated processing of camera trap images is the use of complex deep learning algorithms and processes, usually accessible only to computer scientists. U-Infuse bridges this gap by allowing ecologists to train their own models via a user-friendly GUI. U-Infuse is therefore an important connecting inter-phase between computer science and ecology as it allows field practitioners to undertake tasks usually only understood by and reserved to computer scientists. It represents a significant progression from early studies, which involved ecological practitioners collecting images, manually cataloguing and placing bounding boxes around animals, providing these images to computer scientists to develop domain specific object detectors, which is a major limitation of solutions such as ClassifyMe [20].

Furthermore, U-Infuse may be extended to support other frameworks including YOLOv3 (Redmon and Farhadi 2016), Faster RCNN [42] and Single Shot Detectors (SSDs) [43]. This would offer more options to users, for example YOLOv3 and SSDs are capable of real-time performance, while Faster RCNN provides greater accuracy, but is slower. Incorporating FlickR API support to allow image downloading and sorting within U-Infuse, and integration of the Structural Similarity Index Measure (SSIM) image similarity tool and duplicate remover [31] would also extend its usability in the development of high performance, domain specific deep learning object detectors. Copying, distribution and modification of U-Infuse source code is encouraged. Accordingly, U-Infuse is distributed under the terms of a GNU General Public License (https://www.gnu.org/licenses/gpl-3.0.en.html accessed on 1 April 2021).

## Figures and Tables

**Table 1:** Summary of pretrained models included with U-Infuse installation.

Model Name	Classes	Source
pretrained_COCO	80 classes	MS COCO
Australian_Multi-class	30 classes	FlickR
pig_single_class	Pig	FlickR
striped_hyena_single_class	Dog	FlickR
rhino_single_class	Cat	FlickR

**Table 2:** FiN stands for FlickR and iNaturalist images. Images were downloaded and pre-processed using the procedure described by [31].

Dataset		Description	N^o^ Images	N^o^ Capture Events
Training	Positive Samples	FiN_feral_cats	1000	NA
		AU_Feral_Cat_A_Grade_1	166	NA
		AU_Feral_Cat_A_Grade_2	50	NA
	Negative Samples	FiN_australian_animals	818	NA
		FiN_brushturkey	335	NA
		FiN_deer	340	NA
		FiN_dog	355	NA
		FiN_fox	292	NA
		FiN_koala	309	NA
		FiN_quoll	287	NA
		FiN_random_negatives_feral_cat_version	782	NA
		infusion_AU_Unbalanced_A_Grade_Trap_Fox	56	NA
		infusion_AU_Unbalanced_A_Grade_Trap_Other (koalas, goannas, possums, etc.)	63	NA
		infusion_AU_Unbalanced_A_Grade_Trap_Kangaroo	53	NA
		infusion_AU_Unbalanced_A_Grade_Trap_Feral_dog	53	NA
		infusion_AU_Unbalanced_A_Grade_Trap_Pig	53	NA
		blank_trap	31	NA
Testing	Positive Samples	NE_Gorge_trap_2_day	130	128
		NE_Gorge_trap_2_infrared	403	135
		Wellington Camera Traps (cat)	1347	449
	Negative Samples	NE_Gorge_trap_Dog	64	NA
		NE_Gorge_trap_Kangaroo	20	NA
		NE_Gorge_trap_Blank	64	NA
		NE_Gorge_trap_Fox	64	NA
		NE_Gorge_trap_Pig	87	NA
		NE_Gorge_trap_Other	162	NA

**Table 3:** Performance of felis_catus_detector on images containing cats ranged from 55–75% accuracy when calculated on a per image basis. Performance was significantly better when evaluated on a per capture event basis (73.94–84.44%), which is more useful in for practical purposes. The model performed well on negative samples, with 80–100% of images being correctly classified.

Test Set		Per Capture Event	Per Image
Positive	NE_Gorge_trap_2_day	76.56% (98/128)	75% (98/130)
	NE_Gorge_trap_2_infrared	84.44% (114/135)	66% (266/403)
	Wellington Camera Traps (cat)	73.94% (332/449)	55% (743/1347))
Negative	NE_Gorge_trap_Dog	NA	94% (60/64)
	NE_Gorge_trap_Kangaroo	NA	85% (3/20)
	NE_Gorge_trap_Blank	NA	100% (29/29)
	NE_Gorge_trap_Fox	NA	80% (51/64)
	NE_Gorge_trap_Pig	NA	80% (70/87)
	NE_Gorge_trap_Other	NA	86% (140/162)

## Data Availability

U-Infuse can be accessed and installed by visiting https://github.com/u-infuse/u-infuse (accessed on 1 April 2021). All datasets, and Jupyter notebooks/tutorials can also be accessed on this GitHub page.
